# Advanced modeling of Alzheimer’s disease using human iPSC‐derived microglia and neurons carrying disease‐related mutations

**DOI:** 10.1002/alz.088752

**Published:** 2025-01-03

**Authors:** Berta Sanz Morello, Elin Byman Shatri, Sofie Frandsen, Benjamin Schmid, Kenneth Thirstrup, Bjørn Holst

**Affiliations:** ^1^ Bioneer, Horsholm Denmark

## Abstract

**Background:**

Microglia are the primary immune cells of the brain and represent the main line of defense against brain environmental insults. In recent years, microglia have been implicated in Alzheimer’s disease (AD) pathogenesis by having interconnected yet opposing roles: beneficial as they clear amyloid beta (Aβ) and amyloid plaques, and detrimental as being responsible for synaptic and neuronal loss. These activities are tightly regulated by microglia receptors CD33 and TREM2. Microglial expression of both CD33 and TREM2 is upregulated and correlates with Aβ plaque load in the brain of AD patients, and genome‐wide association studies have associated genetic variants of CD33 and TREM2 with AD progression. Interestingly, humans carrying the polymorphic allele rs12459419(T) of CD33, which results in the loss of exon 2 in the CD33 transcript giving rise to a shorter isoform of CD33 (CD33‐D2), show higher CD33 expression in microglia and decreased Aβ deposition, lowering AD risk. In contrast, the heterozygous variant R47H TREM2 increases AD risk by four‐fold.

**Method:**

We characterized the phagocytic and surveillance activity of human iPSC‐derived microglia carrying R47H TREM2 or Exon 2‐deleted CD33 (CD33^ΔE2^) in monoculture. Moreover, we analyzed how these microglia genotypes impact Aβ accumulation and neuronal integrity in a robust AD in‐vitro model represented by iPSC‐derived neurogenin‐2 (NGN2) neurons carrying the PSEN1 E280A mutation.

**Result:**

iPSC‐derived microglia carrying R47H TREM2 or CD33^ΔE2^ show altered phagocytic and migratory activities. When co‐cultured with NGN2 neurons carrying PSEN1 E280A, R47H TREM2 microglia and CD33^ΔE2^ microglia affect Aβ40 and Aβ42 levels and neuronal integrity.

**Conclusion:**

Insights into how CD33^ΔE2^ and R47H TREM2 mutations regulate microglial activity in an AD context can help to characterize them as potential therapeutic targets to arrest AD progression.

